# Pediatric patient presenting with bumps within scar

**DOI:** 10.1016/j.jdcr.2024.02.019

**Published:** 2024-03-04

**Authors:** Katlyn Smaha, Victoria M. Madray, Aubrey A. Hess, Morgan W. Thakore

**Affiliations:** a Medical College of Georgia at Augusta University, Augusta, Georgia; b Department of Dermatology, Medical College of Georgia at Augusta University, Augusta, Georgia

**Keywords:** locus minoris resistentiae, molluscum contagiosum, pediatrics, scar

## History

**Fig 1:**
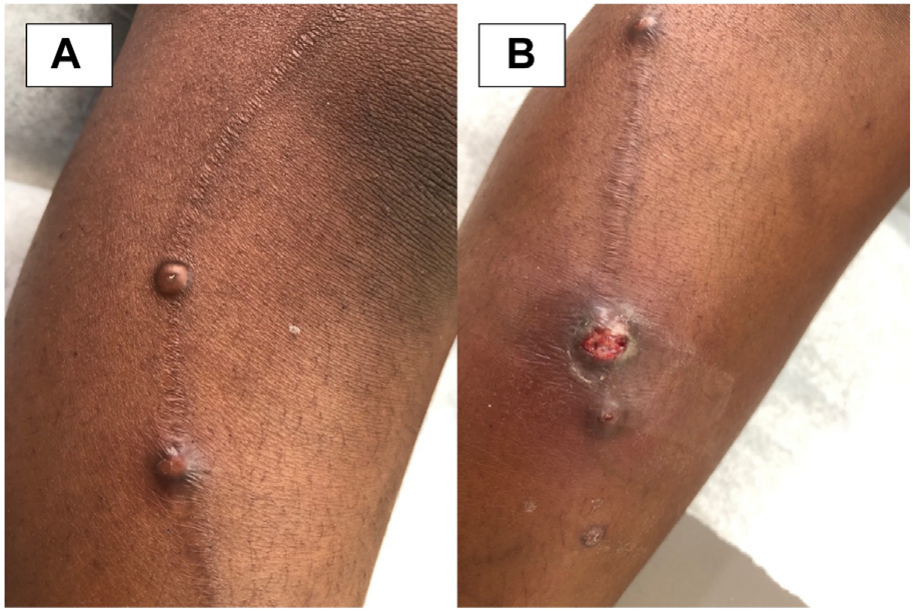

**Fig 2:**
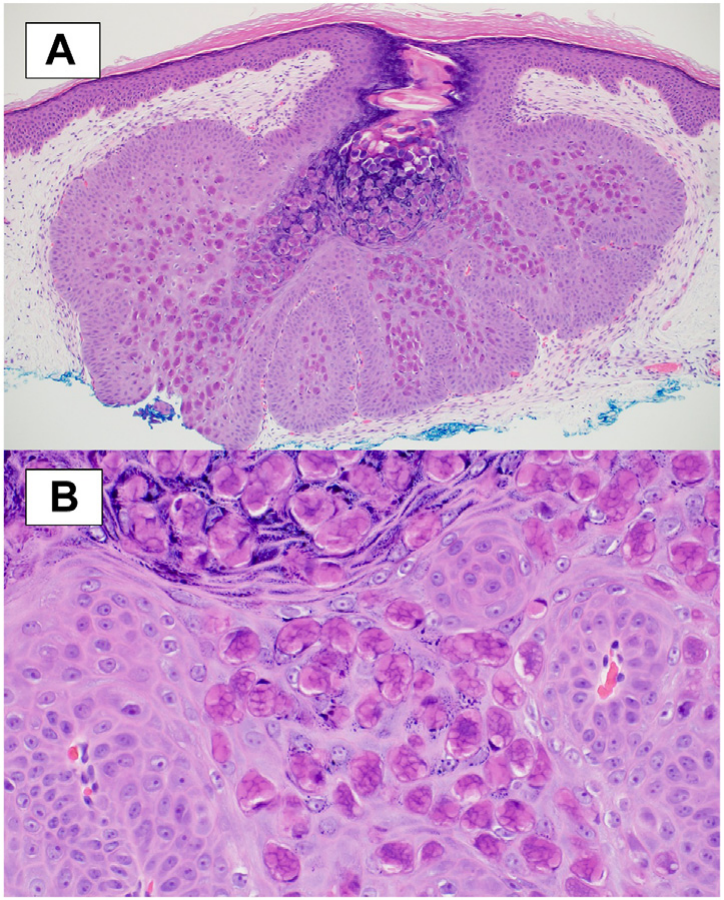

An 8-year-old female with no significant medical history presented with new, rapidly enlarging bumps arising within a cicatrix. The cicatrix formed after an injury 1 year prior and was unchanged until bumps began forming 2 weeks before presentation. The lesions were pruritic and drained white material. The patient’s mother reported constant scratching, which led to excoriation of one of the lesions. Physical exam revealed flesh-colored, umbilicated, oozing papules localized within a cicatrix (Fig 1). Shave biopsy was obtained, and histological findings are shown (Fig 2).

**Question 1: What is the most likely diagnosis?**

A. Arthropod bite
B. Keloid
C. Molluscum contagiosum (MC)
D. Perforating dermatosis
E. Spitting suture

**Answers:**

A. Arthropod bite – Incorrect. Commonly present as erythematous, sometimes edematous, papules. If there is more than 1 bite, they are likely to be in a scattered distribution.
B. Keloid – Incorrect. Present as firm, rubbery nodules in an area of prior skin injury and typically develop slowly over months. In contrast to hypertrophic scars, keloidal tissue grows beyond the initial site of injury and involves the entire scar.
C. MC – Correct. MC is an acute infection caused by molluscum contagiosum virus that is transmitted via skin-skin contact. It is common in the pediatric population, with a reported prevalence between 5.1% and 11.5% in children aged 0 to 16.^1^ There is increased risk of infection in immunosuppressed patients.^1^ Typical lesions are dome-shaped, umbilicated, and white, pink, or flesh-colored papules. Lesions are usually asymptomatic but may be painful or pruritic. Histopathology classically shows epidermal hyperplasia with large intracytoplasmic eosinophilic inclusion bodies within keratinocytes, known as Henderson-Paterson bodies.^2^
D. Perforating dermatosis – Incorrect. Represents a group of skin disorders characterized by pruritic follicular hyperkeratotic papules on the hair-bearing extremities of adults. Lesions are occasionally umbilicated.
E. Spitting suture – Incorrect. Represents a common complication of dermatologic surgery that typically develops 2 weeks to 3 months following closure of cutaneous defect with buried sutures. Manifest as small, erythematous papules along the surgical site, sometimes with visible or palpable threads on the surface.

**Question 2: What common dermatologic disorder has been proposed as a risk factor for this condition?**

A. Psoriasis
B. Rosacea
C. Acne vulgaris
D. Atopic dermatitis (AD)
E. Vitiligo

**Answers:**

A. Psoriasis – Incorrect. While MC has been reported in patients on biologic therapy for psoriasis, it is the immunosuppressive state induced by the antipsoriatric treatment that increases the susceptibility to the development of MC.
B. Rosacea – Incorrect. Has not been associated with increased risk of MC.
C. Acne vulgaris – Incorrect. Has not been associated with increased risk of MC. Given the overlapping affected age group and similar clinical presentation, MC may be mistaken for acne. Acne more commonly affects adolescents and young adults, rather than young children.
D. AD – Correct. Has been proposed as a risk factor for MC since it disrupts the skin barrier and immunity function.^2^ Patients with AD may be more likely to spread MC via self-inoculation, leading to more lesions that last longer. Our patient did not have a history of eczema. However, even in patients without AD, eczematous changes may develop around mollusca which predisposes to increased skin irritation and pruritus.^2^ An inflammatory reaction secondary to scratching may result. Alternatively, the inflammatory reaction may represent the “beginning of the end” sign or Meyerson phenomenon.^3^ This often precedes resolution of the virus, rather than represents secondary bacterial superinfection. Given that our patient’s lesions had been present for 2 weeks, it is unlikely that they were already resolving. Lesions of molluscum contagiosum virus usually resolve in 6 to 12 months.^1^^,^^2^
E. Vitiligo – Incorrect. Has not been associated with increased risk of MC. However, treatment with immunosuppressive medications and ointments may predispose to infection with MC.

**Question 3: What underlying theory explains the presence of these lesions within a cicatrix?**

A. Fibroblast proliferation and excess collagen production
B. Koebnerization
C. Pseudo-koebnerization
D. Locus minoris resistentiae
E. Granulomatous inflammation

**Answers:**

A. Fibroblast proliferation and excess collagen production – Incorrect. Consistent with keloid formation.
B. Koebnerization – Incorrect. Describes the development of new lesions of a pre-existing skin condition on areas of cutaneous injury. Commonly seen in psoriasis, lichen planus, and vitiligo.
C. Pseudo-koebnerization – Incorrect. Occurs when infections arise in an area of trauma due to the transfer of infectious agents into the damaged skin, usually through scratching. Although pseudo-koebnerization of MC has been reported in the literature, this patient’s wound healed and formed a scar 1 year prior to molluscum lesions appearing.^4^
D. Locus minoris resistentiae – Correct. Locus minoris resistentiae in Latin means “place of lesser resistance.” The term is given when an area is more susceptible to disease than others; infection occurs via the path of least resistance. Therefore, areas of injury or trauma may be weakened and more susceptible to viral presentation.^5^ Given MC has a latency period of 1 to 50 days, appearance within the cicatrix after 1 year cannot be explained by self-inoculation but rather molluscum contagiosum virus as a part of locus minoris resistentiae.^2^^,^^5^ While a previous area of trauma may be more susceptible to lesion formation, there are no reports to suggest an inability to clear the infection once formed.^5^ Therefore, these lesions may heal by a similar spontaneous mechanism as other MC infections, which may involve Meyerson phenomenon.^3^
E. Granulomatous inflammation – Incorrect. Consistent with a spitting suture or other foreign body reaction. Histopathology demonstrates a focal collection of epithelioid cells or histiocytes and multinucleated giant cells.

## Conflicts of interest

None disclosed.
